# Analyzing the Transcriptome Profile of Human Cumulus Cells Related to Embryo Quality via RNA Sequencing

**DOI:** 10.1155/2018/9846274

**Published:** 2018-08-01

**Authors:** Qiwei Liu, Junhui Zhang, Huihui Wen, Yun Feng, Xunyi Zhang, Huifen Xiang, Yunxia Cao, Xiaowen Tong, Yazhong Ji, Zhigang Xue

**Affiliations:** ^1^Department of Gynecology and Obstetrics, Affiliated Tongji Hospital, Tongji University, Shanghai, China; ^2^Department of Gynecological Minimal Invasive Center, Beijing Obstetrics and Gynecology Hospital, Capital Medical University, Beijing, China; ^3^Translational Center for Stem Cell Research, Tongji Hospital, Department of Regenerative Medicine, Tongji University School of Medicine, Shanghai, China; ^4^Reproductive Medicine Center, Tongji Hospital, Tongji University School of Medicine, Shanghai, China; ^5^Reproductive Medicine Center, The First Affiliated Hospital of Anhui Medical University, Hefei, China

## Abstract

Selecting excellent oocytes is required to improve the outcomes of in vitro fertilization (IVF). Cumulus cells (CCs) are an integral part of the oocyte maturation process. Therefore, we sought to identify differentially expressed genes in CCs to assess oocyte quality and embryo development potential. We divided the participants' embryos into the high-quality embryo group and low-quality embryo group by the information including age, body mass index, and the levels of luteinizing hormone, follicle-stimulating hormone, estradiol, and progesterone. We analyzed a total of 7 CC samples after the quality control in RNA sequencing. We found that 2499 genes were unregulated and 5739 genes were downregulated in high-quality embryo group compared to the low-quality embryo group (*Padj *< 0.05). Interestingly,* MSTN, CTGF, NDUFA1, VCAN, SCD5, *and* STAR* were significantly associated with the quality of embryo. In accordance with the results of RNA sequencing, the association of the expression levels of* MSTN, CTGF, NDUFA1, VCAN, SCD5, *and* STAR* with the embryo quality was verified by quantitative reverse-transcription polymerase chain reaction (RT-qPCR) in 50 CC samples. Despite the small sample size and lack of validation in animal models, our study supports the fact that differential gene expression profile of human CCs, including* MSTN, CTGF, NDUFA1, VCAN, SCD5, *and* STAR*, can serve as potential indicator for embryo quality.

## 1. Introduction

It is well known that the interaction of oocytes with their surrounding cumulus cells (CCs) is essential for oocyte development, and CCs provide the oocytes with metabolic support and microenvironment signals [1]. Researchers have suggested that significant gene expression profiles in CCs could be related to the quality of oocytes and could be used to assess embryo viability, which suggests that differentially expressed genes in CCs play a vital role in the process of oocyte maturation [2–4]. Moreover, the ability to assess oocyte quality is critical to identifying viable embryos after fertilization [5]. Despite major advancements in assisted reproductive technology (ART), the assessment of embryo potential is limited, which can increase pregnancy failure and multiple pregnancies [6]. Currently, strategies for predicting embryo quality are based on embryo morphological parameters; however, prolonged culture may have negative effects on embryo development, including epigenetic modifications and adverse fetal outcomes [7, 8]. As oocyte and ovarian cryopreservation are increasingly used for older women or women with premature ovarian failure, accurate assessment of oocyte quality has attracted more attention. Therefore, identifying a reliable oocyte assessment method can be advantageous for selecting high-quality embryos.

To our knowledge, this study is the first to use single-cell RNA sequencing (RNA-Seq) technology to analyze transcriptome gene expression of human CCs isolated from oocytes. In this study, we compared the transcriptome profiles of CCs from high-quality embryos with low-quality embryos and then evaluated the relationship between significantly differentially expressed genes and embryo outcomes. Hence, we were able to find a more precise approach to predicting oocyte quality and embryo development potential through transcriptome profile analysis.

## 2. Materials and Methods

### 2.1. Study Population

In our study, we recruited 30 women, all of whom were undergoing ART at Anhui First People's Hospital in Anhui, China, from March 2017 to June 2017, and were willing to donate CCs. Via medical chart review, we confirmed that the participants' spouses had no infertility problems: normal semen quality, including semen volume (≥1.5ml), sperm concentration (≥1.5×106 /mL), pH≥7.2, sperm motility (≥32%), survival rate of spermatozoa (> 58%), and liquefying time (≤60 min) (WHO laboratory manual for the examination and processing of human semen). Ovarian stimulation for all participants had been carried out according to the standard long protocol. 0.8 mg/d of GnRH-agonist (gonadotropin-releasing hormone) was given by intramuscular injection from the 21st day of the run-in cycle for 14 days. After menstruation and hormone (E2<50 pg/ml) which meant pituitary block, 300IU of Gn was given intranasally until more than 2 follicles with a diameter of 18mm were observed through B-ultrasonography. Then we stop Gn, and HCG (human chorionic gonadotropin) was administered at a dose of 1000 IU. After 36 hours, follicles could be taken out. Patients' demographic and clinical characteristics such as age, body mass index (BMI), and luteinizing hormone (LH), follicle-stimulating hormone (FSH), estradiol (E2), and progesterone levels were recorded. The study protocol was approved by the Research Ethics Committee of Anhui First People's Hospital (No: 2014008) and conducted in accordance with approved institutional guidelines. All participants gave written informed consent.

### 2.2. Isolation of CCs and Assessment of Embryo Quality

Cumulus cell-oocyte complexes (COCs) were isolated via ultrasound-guided vaginal puncture and washed in phosphate-buffered saline (PBS). Based on the Smart-seq2 protocol, one sample included 10 CCs, which were collected randomly from one oocyte and transferred to a tube with lysis buffer for RNA-Seq. 75 samples were totally collected from 30 participants. 15 samples from these samples were randomly selected to be used in RNA-Seq experiments. In the RNA-Seq procedure, only 7 samples passed the quality control and were further analyzed. And the remaining samples were prepared for quantitative reverse-transcriptase polymerase chain reaction (RT-qPCR).

Oocytes were fertilized via intracytoplasmic sperm injection. After 16-18 hours of fertilization, the oocytes were assessed via stereomicroscopy and categorized as follows: 0PN (pronuclei not visible, unfertilized); 1PN (fewer than 2 pronuclei, abnormally fertilized); 3PN (more than 2 pronuclei, abnormally fertilized); or 2PN (2 pronuclei, normally fertilized).

We used morphological strategies to assess embryo quality on day 3. High-quality embryos were identified using the following characteristics: 8-10 cells on the third day of development, less than 20% fragmentation, symmetric blastomeres, and no multinucleation and zona pellucida dysmorphisms. Embryos not meeting all the aforementioned conditions were considered low-quality embryos.

### 2.3. Library Construction and Sequencing

We referred to the Smart-seq2 protocol for all RNA-Seq experiments [9]. Cells were washed twice with 1X PBS before being placed in lysis buffer. RNA was isolated from cells and converted into cDNA. Library construction was performed following the Smart-seq2 protocol, and sequenced reads that contained polyA tails, low-quality regions, and adapters were prefiltered before mapping. cDNA was sheared into 100- to 150-bp short fragments according to the manufacturer's instructions. Libraries were pooled and sequenced on Illumina HiSeq2500 sequencers. Data normalization was carried out by transforming mapped transcript reads to fragments per kilobase of transcript per million mapped reads. Genes with fragments per kilobase of transcript per million mapped reads > 0.5 were retained for analysis.

### 2.4. RNA Extraction and Validation of RNA-Seq Data by RT-qPCR

According to the Smart-seq2 protocol, RNA was extracted from 8 to 10 CCs per sample using oligo-Dt30VN primer and dNTP at 72°C and then was reverse transcribed into cDNA. After the first-strand reaction, cDNA was amplified. First, we purified cDNA by using AMPure XP beads and 80% ethyl alcohol. Second, significantly differentially expressed genes were confirmed by RT-qPCR (208054, QIAGEN, Germany). The primers used in this study are shown in Table 1. All the genes were normalized to glyceraldehyde-3-phosphate dehydrogenase (GAPDH). RT-qPCR was performed in a total reaction volume of 25ul, including 1 ul cDNA (1 ng/ul), 10 ul 2x SYBR green PCR master mix, 0.1 ul QN ROX reference dye, 1 ul forward primer (10 mmol/L), 1 ul reverse primer (10 mmol/L), and 6.9 ul RNase-free water. PCR initial activation was achieved by heating samples to 95°C for 2 min, followed by a total of 40 cycles of denaturation at 95°C (5 s) and 60°C (30 s) in a total of 40 cycles.

### 2.5. Gene Expression Analysis and Statistics

Differential gene expression was considered significant if the adjusted* P* (*Padj*) < 0.05.* Padj* was calculated via the Benjamini-Hochberg method to exclude false-positive results. All clinical parameters were expressed as mean ± standard deviation depending on the distribution. Gene ontology was analyzed via hypergeometric distribution. Heatmaps were constructed using R software. The Kyoto Encyclopedia of Genes and Genomes (KEGG) was analyzed in our study. Differences in clinical outcomes between high-quality embryo group and low-quality embryo group were detected via a t-test and Mann–Whitney test using SPSS software, version 20.0. All data were normally distributed (Shapiro-Wilk, Sig. > 0.05; Levene's test, Sig. >0.05), so the independent-samples t-test was used to analyze the scores of the groups.

## 3. Results

### 3.1. Clinical and Biochemical Characteristics of Patients

The clinical and biochemical characteristics of 30 patients who participated in our study are shown in Table 2, including 17 participants in poor-quality group and 13 participants in high-quality group. There was no statistically significant difference between the two groups in age, BMI, LH, FSH, E2, and progesterone levels.

### 3.2. RNA-Seq Analysis

**Figure 1:**
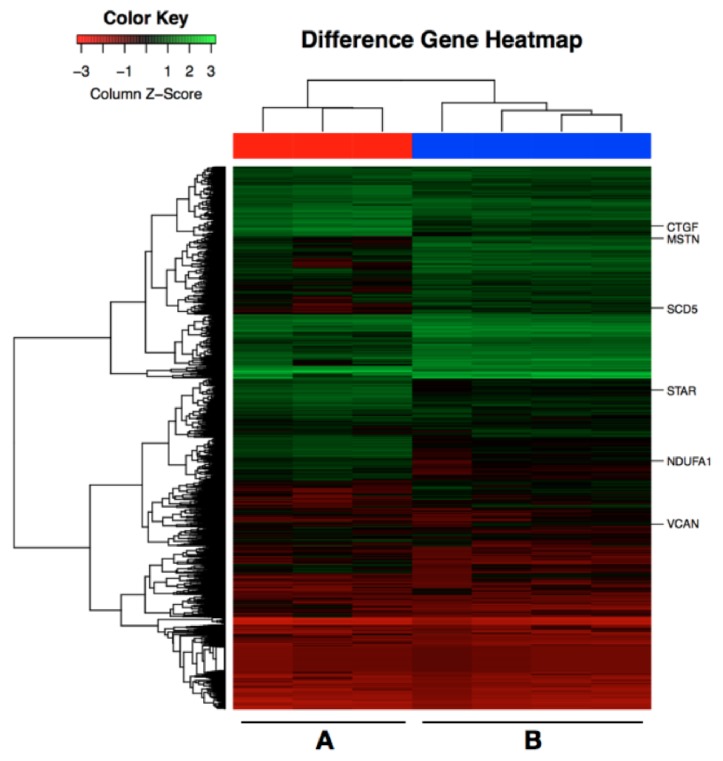
**Heatmap of differentially expressed genes in CCs from high-quality embryos and low-quality embryos.** Red corresponds to downexpressed genes, and green corresponds to upexpressed genes. Group A, cumulus cells from high-quality oocytes on day 3 (n=3). Group B, cumulus cells from low-quality oocytes on day 3 (n=4).

**Figure 2:**
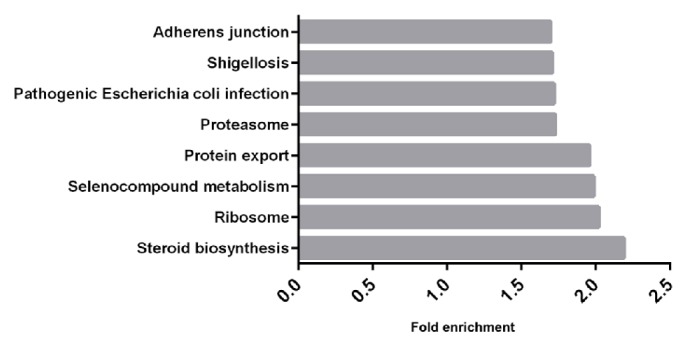
Differential pathways analyzed by KEGG. Fold enrichment is the P value hypergeometric test adjusted by the multiple test adjustment (-log10 P value),* Padj *< 0.05.

**Figure 3:**
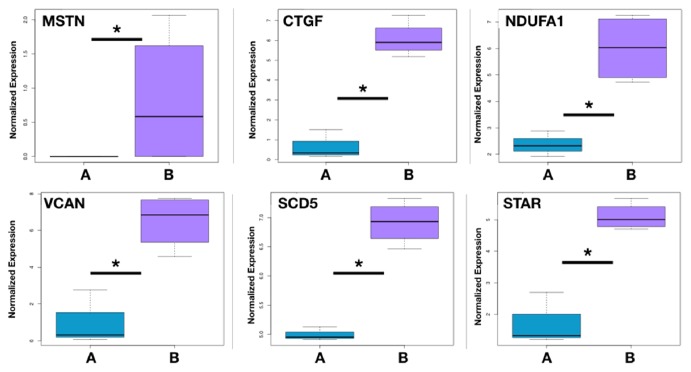
**Differentially expressed genes in CCs from high-quality embryo compared with CCs from low-quality embryo as deduced from data derived from RNA sequencing studies**. Group A, cumulus cells from high-quality oocytes on day 3 (n=3). Group B, cumulus cells from low-quality oocytes on day 3 (n=4). MSTN/GDF8: myostatin; CTGF: connective tissue growth factor; NDUFA1: ubiquinone oxidoreductase subunit A1; VCAN: versican; SCD5: stearoyl-CoA desaturase 5; STAR: steroidogenic acute regulatory protein. ^*∗*^Padj<0.05.

7 CCs samples were selected to be analyzed via RNA-Seq, which were divided into 2 groups. CCs in group 1 were picked up from high-quality oocytes, while CCs in group 2 were from low-quality oocytes (Figure 1). The minimum fold change was 2.0. A total of 2499 upregulated genes and 5739 downregulated genes in the high-quality embryo group were compared to the low-quality group (*Padj *< 0.05). KEGG analysis revealed that differentially expressed genes in CCs from high-quality oocytes were mainly involved in the steroid biosynthesis pathway, ribosome pathway, selenocompound metabolism pathway, protein export pathway, proteasome pathway, pathogenic Escherichia coli infection pathway, shigellosis pathway, and adherens junction pathway (Figure 2). We found 6 genes including myostatin (*MSTN/GDF8*), connective tissue growth factor (*CTGF*), ubiquinone oxidoreductase subunit A1 (*NDUFA1*), versican (*VCAN*), stearoyl-CoA desaturase 5(*SCD5*), and steroidogenic acute regulatory protein (*STAR*). From RNA-Seq outcome was significantly higher expression in CCs from oocytes yielding poor embryos (Figure 3).

### 3.3. RT-qPCR Analysis

**Figure 4:**
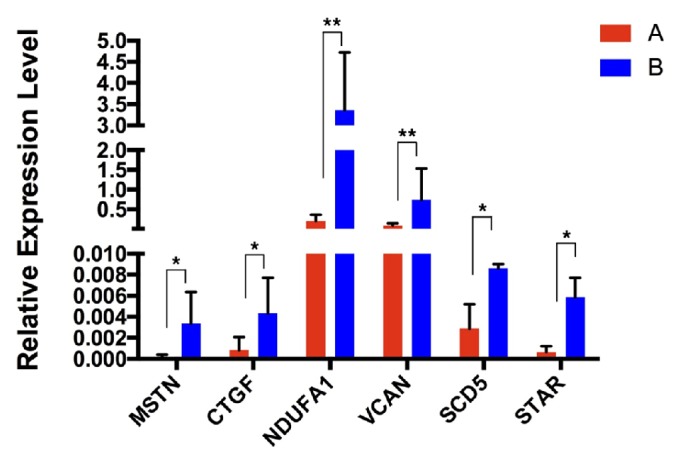
**Differentially expressed genes in CCs from high-quality embryo compared with CCs from low-quality embryo by RT-PCR**. Group A, gene expression levels in CC samples from high-quality oocytes (n=20). Group B, gene expression levels in CC samples from low-quality oocytes (n=40). ^*∗*^P value < 0.05; ^*∗∗*^P value< 0.01.

**Figure 5:**
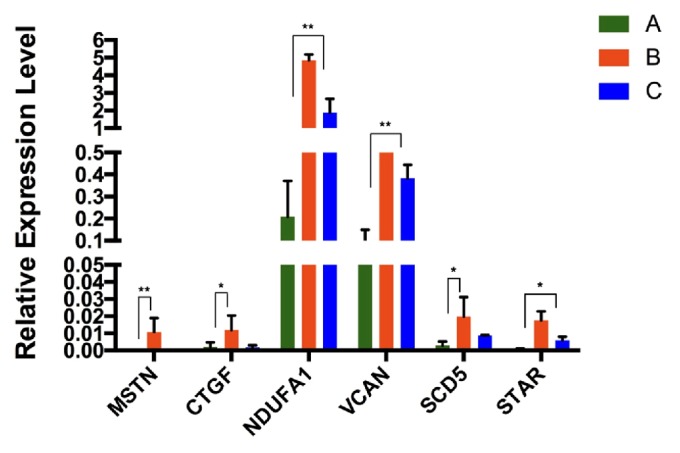
**Differentially expressed genes in CCs from high-quality embryo compared with CCs from low-quality embryo and unfertilized embryo by RT-PCR**. Group A, gene expression levels in CCs from oocytes yielding high-quality embryos (n=20). Group B, gene expression levels in CCs from oocytes yielding low-quality embryos (n=20). Group C, gene expression levels in CCs from oocytes failing to form embryos (n=20). ^*∗*^P value < 0.05; ^*∗∗*^P value < 0.01. Gene expression levels in group B and group C were both higher than the levels in group A.

There were 60 CC samples collected from 30 women divided into 2 groups according to embryo quality level to be analyzed via RT-qPCR (Figure 4), including 20 CC samples from oocytes yielding high-quality embryos and 40 CC samples from oocytes yielding poor-quality embryos and from oocyte failing to form embryo. The results of RT-qPCR suggested that those 6 genes had significantly higher expression in CCs from high-quality oocytes compared with CCs from low-quality oocytes. Then we also divided 40 CC samples from poor-quality group into 2 groups that are 20 CC samples from oocytes yielding low-quality embryos and 20 CC samples from oocytes failing to fertilize after 16-18 h, respectively (Figure 5). The results corresponded to the RNA-Seq outcomes, which confirmed that those genes were differentially expressed in CCs from oocytes yielding low-quality embryos.

## 4. Discussion

Our findings show that some genes that are differentially expressed in CCs are related to the embryo quality and could be genetic markers for predicting embryo potential. We used single-cell RNA-Seq to identify differentially expressed genes in CCs from both high-quality and low-quality oocytes depending on their fertilization outcomes. Meanwhile, the results from RNA-Seq also showed that some differential pathways may demonstrate oocyte development, such as the steroid biosynthesis pathway, ribosome pathway, selenocompound metabolism pathway, protein export pathway, proteasome pathway, pathogenic Escherichia coli infection pathway, shigellosis pathway, and adherens junction pathway. Thus, we could infer that failed fertilization of oocytes was mainly related to abnormal levels of steroid hormones, growth factors, and adherens junction pathway.

Steroid hormones pathway participate in the process of folliculogenesis and oogenesis, such as LH, FSH, progesterone, estrogen, and androgen [10, 11]. For example, FSH stimulates CCs proliferation and the secretion of liquor folliculi, which are vital for follicular maturation [12]. LH can stimulate the synthesis of regulate hyaluronic acid and cumulus maturation factors, which can induce CCs expansion and germinal vesicle breakdown of oocytes [13]. Under interaction of FSH with LH, estrogen and androgen are produced in the ovary, which regulates female fertility [14]. Therefore, an imbalance in steroid hormones can impede folliculogenesis and oogenesis. Insulin growth factors (*IGF*s), epidermal growth factors (*EGF*s), and transforming growth factors (*TGF*s) are all important factors for regulating follicular development [15–17]. Dysfunction of the protein export process can affect the production of those factors, which are composed of proteins. Junction CCs and oocytes were the key bridge to conveying growth factors and hormone regulation [18]. Thus, the dysfunction of the adherens junction could cause growth arrest of oocyte.

We used RT-qPCR to verify some differentially expressed genes identified via RNA-Seq, including* MSTN, CTGF, NDUFA1, VCAN, SCD5, *and* STAR*, which may be prognostic markers of oocyte quality. COCs are vital part of follicles that regulate follicle growth by secreting factors and communicating via gap junctions [19]. Oocytes regulate CCs proliferation and the sensitivity capacity of steroid hormones, while CCs regulate oocyte maturation, such as growth, meiosis, and ovulation [20, 21]. Therefore, the expression profiles of genes in CCs could be used as genetic markers to predict the quality of oocytes and the development of embryos. The process we selected for differentially expressed genes, which may predict the quality of embryo, was based on the function of genes in CCs, including connecting gap communication, stimulating oocyte growth, meiosis and ovulation, and regulating CCs proliferation. Finally, we found 6 differentially expressed genes that were closely related to the oocyte maturation process and could be differentially expressed between high-quality embryos and low-quality embryos.

Previous studies have shown that* GDF8/MSTN* downregulates the expression of* PTX3*, which is produced by CCs and regulates cumulus expansion, ovulation, and in vivo fertilization [22, 23]. In mice, the absence of* PTX3* was related to abnormality of the cumulus oophorus. On the other hand, overexpressed* MSTN *decreased the expression profile of the LH receptor and progesterone, whereas the expression of FSH receptor and estradiol was increased [24]. Therefore,* MSTN *is associated with the oocyte microenvironment and proliferation of CCs. Our findings showed that the expression of* MSTN* was significantly higher in CCs from low-quality embryos than those from high-quality embryos, which suggests that the main reasons for failed embryo development were poor microenvironment and CC dysfunction.* MSTN *expression levels were also significantly higher in CCs from abnormal pronuclei than in CCs from high-quality embryos. The results showed that* MSTN *was closely associated with the process of fertilization and embryo development.* CTGF* is in the same pathway with* MSTN* and regulates* MSTN* expression positively and also participates in COC development [25]. In our study,* CTGF* was also upregulated in CCs from low-quality embryos compared with those from high-quality group. However, there was no difference between the gene profiles of CCs from high-quality embryos and those from abnormal pronuclei, which suggests that* CTGF* did not participate in the fertilization process but was important for embryo development.

*NDUFA1* is a mitochondrial antioxidant that is expressed in response to oxidative stress and endoplasmic reticulum. Previous reports showed that higher expression of* NDUFA1* in CCs could accelerate cell aging in mice [26].* VCAN *participated in CCs development and was also related to maternal age [2, 27]. In our study,* NDUFA1* and* VCAN *were significantly upregulated in CCs from oocytes yielding low-quality embryos and from oocytes failing in fertilization, which inhibited the development of COCs.

*SCD* was expressed both in CCs and in oocytes, and it provides unsaturated fatty acids to the oocyte [28]. Researchers previously localized* SCD* mRNA via in situ hybridization in rats and found that* SCD* was associated with human chorionic gonadotropin, FSH, corpus luteum, and granulose cell proliferation [29, 30].* STAR *is involved in steroidogenic activity of ovaries and is expressed in CCs via the LH surge [31, 32].* STAR* was showed to be involved in the mitogen-activated protein kinase activity that regulates steroidogenesis in COCs [33]. Both* SCD5* and* STAR* were associated with steroid hormone, and this association is vital for cross interaction between oocytes and CCs. In our study, both RNA-Seq and RT-qPCR revealed higher expression of the above 6 genes in CCs from oocyte yielding low-quality embryos. It is possible that COCs had a poor response to the hormone reaction, which resulted in poor embryo formation. However,* SCD5* was differentially expressed between CCs from good embryo and from poor embryo but not from abnormal pronuclei. The expression of* STAR* was higher in CCs from abnormal pronuclei than in CCs from oocyte yielding high-quality embryos, which suggests that* STAR* participates in embryo development from the beginning of pronuclei formation.

In conclusion, we discovered that the expression profiles of certain genes in CCs are associated with oocyte quality and embryo development under IVF. Meanwhile, we realized the limitations in the present work. The sample size was relatively small in our experiment mainly due to the difficulty in collecting human oocytes for the specific research. At present, we are attempting to establish such a clinical sample library to expand the sample size which will facilitate our future research. Of course, we screened the genes at the transcription level, which did not reflect their expression at protein level, posttranslational modification, etc. In the future, we would like to validate the biological functions of these genes by characterizing their expression at different levels and revealing the molecular mechanism in gain-of-function and/or loss-of-function animal models. Nevertheless, examining the transcription level of the genes that we characterized in this work holds the potential to serve as a novel noninvasive technique to assess oocyte quality and embryo development.

## Figures and Tables

**Table 1:** Primers used for qRT-PCR.

**cDNA**	**Sense Primer (5'-3')**	**Antisense Primer (5'-3')**
**MSTN**	ACTGGTGTGGCAAGTTGTCT	TGGACCAGCAACAATCAGCA
**CTGF**	GAGAGTCCTTCCAGAGCAGC	CATAGTTGGGTCTGGGCCAA
**NDUFA1**	GCGTACATCCACAGGTTCAC	ACTCCAGAGATGCGCCTATC
**SCD5**	ATCTTCGAGTGGTCCAGGGA	TGACGTCAAGCTTTCTCCCC
**VCAN**	TGACTGTGGATGGGGTTGTG	GCGTCACACTGCTCAAATCC
**STAR**	TCCTTGACCCCTTCCTTTGC	GCAGCATTGTTTCCTGGCAA
**GAPDH**	CCAGGGCTGCTTTTAACTCTG	TGACAAGCTTCCCGTTCTCA

MSTN, myostatin; CTGF, connective tissue growth factor; NDUFA1, ubiquinone oxidoreductase subunit A1; VCAN, versican; SCD5, stearoyl-CoA desaturase 5; STAR, steroidogenic acute regulatory protein; GAPDH, glyceraldehyde-3-phosphate dehydrogenase.

**Table 2:** Clinical and biochemical characteristics of patients.

	Poor-quality group	High-quality group	P value
	(n=17)	(n=13)	
Age (yr)	34.50±7.0	39.85±5.5	0.053
BMI (kg/m^2^)	21.98±1.6	21.80±2.5	0.854
E2 (pmol/L)	287.99±156.3	214.91±49.16	0.522
LH (IU/L)	8.18±2.9	7.33±1.7	0.398
FSH (IU/L)	3.55±2.3	3.46±1.9	0.919
progesterone	1.37±1.0	2.34±0.8	0.075

BMI, body mass index; FSH, follicle-stimulating hormone; LH, luteinizing hormone; E2, estradiol.
